# Clinical verification of the relationship between smoking and the immune microenvironment of breast cancer

**DOI:** 10.1186/s12967-019-1773-y

**Published:** 2019-01-07

**Authors:** Koji Takada, Shinichiro Kashiwagi, Yuka Asano, Wataru Goto, Katsuyuki Takahashi, Hisakazu Fujita, Tsutomu Takashima, Shuhei Tomita, Kosei Hirakawa, Masaichi Ohira

**Affiliations:** 1 Department of Breast and Endocrine Surgery, Osaka City University Graduate School of Medicine, 1-4-3 Asahi-machi, Abeno-ku, Osaka, 545-8585 Japan; 2 Department of Pharmacology, Osaka City University Graduate School of Medicine, 1-4-3 Asahi-machi, Abeno-ku, Osaka, 545-8585 Japan; 3 Department of Scientific and Linguistic Fundamentals of Nursing, Osaka City University Graduate School of Nursing, 1-5-17 Asahi-machi, Abeno-ku, Osaka, 545-0051 Japan; 4 Department of Surgical Oncology, Osaka City University Graduate School of Medicine, 1-4-3 Asahi-machi, Abeno-ku, Osaka, 545-8585 Japan

**Keywords:** Breast cancer, Smoking, Tumor-infiltrating lymphocytes, Tumor microenvironment, Immune response, Brinkman index

## Abstract

**Background:**

The immune tumor microenvironment (iTME) is thought to affect the response to chemotherapy, and tumor-infiltrating lymphocytes (TILs) are often used as an indicator to evaluate the iTME. Smoking is involved in carcinogenesis, the relationship between smoking and the iTME of lung cancer has been reported. We hypothesized that smoking would affect the iTME of breast cancer and aimed to examine this relationship based on the amount of pre-diagnosis smoking and the subsequent effects on treatment response and prognosis.

**Methods:**

This retrospective study evaluated data from 149 patients who underwent preoperative chemotherapy for triple-negative or HER2-enriched breast cancer. TILs were assessed in biopsy specimens at diagnosis. The data of all patients were used to calculate each patient’s smoking amount based on pack-years.

**Results:**

Relative to the low smoking group, the high smoking group had a significant greater TILs density (p = 0.043) and a significantly better pathological complete response (pCR) rate (p = 0.042). However, there was no significant difference according to smoking amount in disease-free survival (p = 0.114) or overall survival (p = 0.347).

**Conclusions:**

Smoking may influence the iTME, with an activated iTME being associated with pCR rate. Therefore, controlled activation of the microenvironment in this setting may help improve patients’ prognosis.

**Electronic supplementary material:**

The online version of this article (10.1186/s12967-019-1773-y) contains supplementary material, which is available to authorized users.

## Background

The immune tumor microenvironment (iTME) is thought to affect the response to chemotherapy, and tumor-infiltrating lymphocytes (TILs) are often used as an indicator to evaluate the iTME [1–3]. Many studies have revealed that a high TILs density in breast cancer is associated with good therapeutic effects, such as pathological complete response (pCR), prolonged disease-free survival (DFS), and prolonged overall survival (OS) [4, 5]. It became commonly known that affect TILs density in breast cancer is the cancer subtype, with many reports indicating that a high TILs density is associated with high-risk subtypes, such as triple-negative breast cancer (TNBC) and human epidermal growth factor receptor 2-enriched breast cancer (HER2BC) [6, 7]. In recent years, it has also been reported that special genes affect TILs, and it is also important to examine the relationship between genes and the iTME [8, 9].

Smoking is involved in the genesis of many carcinomas, including breast cancer [8], with the carcinogenic substances in tobacco smoke causing chronic inflammatory conditions in the microvessels [10, 11]. Recent studies have also indicated that the iTME is deeply involved in carcinogenesis and that chronic inflammation promotes this process [12, 13]. The relationship between smoking and the iTME of lung cancer has been reported [5, 14], although no reports have examined the relationship between smoking and the iTME of breast cancer. Therefore, we hypothesized that smoking would affect the iTME of breast cancer and aimed to examine this relationship based on the amount of pre-diagnosis smoking and the subsequent effects on treatment response and prognosis.

## Methods

### Patient background

This retrospective study evaluated data from 149 patients who underwent preoperative chemotherapy (POC) for resectable TNBC or HER2BC between February 2007 and December 2017 at the Osaka City University Hospital. All patients were questioned regarding their smoking history at the initial visit (cigarettes smoked per day and years of smoking), and the data were used to calculate each patient’s smoking amount based on pack-years (Table 1). The breast cancers were diagnosed pathologically and classified according to subtype based on the immunohistochemical expression of estrogen receptor (ER), progesterone receptor (PgR), human epidermal growth factor receptor 2 (HER2), and K-i67. Cases were defined as either HER2BC (ER−, PgR−, and HER2+) or TNBC (ER−, PgR−, and HER2−).

**Table 1:** Pack-years of smoking

To calculate smoking pack-years: Divide the number of cigarettes smoked per day by 20 (the number of cigarettes in a pack) Then multiply by the number of years smoked	
ex. 1	(70 cigarettes/day ÷ 20 cigarettes/pack) × 10 years = 35 pack-years
ex. 2	(35 cigarettes/day ÷ 20 cigarettes/pack) × 20 years = 35 pack-years
ex. 3	(20 cigarettes/day ÷ 20 cigarettes/pack) × 20 years = 20 pack-years

All patients received a standardized outpatient POC regimen that consisted of four courses of FEC100 (fluorouracil: 500 mg/m^2^, epirubicin: 100 mg/m^2^, and cyclophosphamide: 500 mg/m^2^) every 3 weeks, which was followed by 12 courses of weekly paclitaxel (80 mg/m^2^). The patients with HER2BC also received trastuzumab during the paclitaxel treatment as a weekly dose (2 mg/kg) or tri-weekly dose (6 mg/kg) [15–17]. Staging and therapeutic effect were evaluated using ultrasonography, computed tomography, and bone scintigraphy based on the Response Evaluation Criteria in Solid Tumors [18]. Patients who achieved clinically partial or complete response were categorized as “responders” in the objective response rate (ORR), while patients with clinically stable or progressive disease were defined as “non-responders”. The patients subsequently underwent mastectomy or breast-conserving surgery [19], and the pathological therapeutic effect of the POC was evaluated using the resected specimens. Pathological complete response (pCR) was defined as complete disappearance of the lesion’s invasive components, including the lymph nodes, with or without intraductal components, according to the National Surgical Adjuvant Breast and Bowel Project B-18 protocol [20]. All patients received postoperative radiotherapy delivered to the remnant breast, and the standard postoperative adjuvant therapy was selected based on the cancer subtype. Patients were followed-up after surgery to detect recurrence using physical examinations every 3 months, ultrasonography every 6 months, and computed tomography and bone scintigraphy annually. The DFS interval was calculated from the day of surgery to the first instance of recurrence or death, while OS was calculated from the day of surgery to death.

### Histopathological evaluation of TILs density

Specimens that were used to pathologically diagnose breast cancer (obtained via core needle biopsy or vacuum-assisted biopsy) were used to determine the TILs density. In the present study, TILs were defined as lymphocytes infiltrating within the tumor stroma [21]. The TILs density was calculated as the average from five randomly selected fields, and the results were classified as a score of 3 (> 50%), a score of 2 (11–50%), a score of 1 (≤ 10%), or a score of 0 (absent) (Additional file 1: Fig. S1). Based on previous reports [22, 23], we defined a high TILs density as scores of 2–3 (i.e., > 10%) and a low TILs density as scores of 0–1 (≤ 10%).

### Statistical analysis

All analyses were performed using JMP software (version 11; SAS Institute, Cary, NC). Differences in the study variables were evaluated using the Chi square test or Fisher’s exact test, as appropriate. The Kaplan–Meier method was used to estimate the DFS and OS outcomes, which were compared using the log-rank test. A Cox proportional hazards model was used to calculate hazard ratios (HRs) and 95% confidence intervals (CIs), and multivariable analysis was performed using a Cox regression model and the backward stepwise selection method. Differences were considered statistically significant at p-values of < 0.05.

### Ethics statement

This study was conducted at the Osaka City University Graduate School of Medicine (Osaka, Japan) according to the Reporting Recommendations for Tumor Marker Prognostic Studies (REMARK) guidelines. The study protocol involved a retrospectively written research, pathological evaluation, and statistical analysis plan [24]. The study complied with the provisions of the Declaration of Helsinki, and all patients provided written informed consent for their treatment and data collection. The study’s retrospective protocol was approved by the ethics committee of Osaka City University (#926).

## Results

### Clinicopathological features

The clinicopathological features of the 149 women are listed in Table 2. The median age at surgery was 56 years (range 24–75 years old). The median follow-up duration was 1288 days after surgery (range 13–3615 days). The median tumor diameter was 27.6 mm (range 10.2–98.0 mm) and 98 patients (65.8%) were diagnosed with N1–3 lymph node metastasis based on their imaging results. Sixty-two patients had HER2BC (41.6%) and 87 patients had TNBC (58.4%). Ninety-one patients (61.1%) had a high TILs density and 58 patients (38.9%) had a low TILs density at their diagnosis. One hundred and five patients (70.5%) reported never smoking, and 44 patients (29.5%) reported a median smoking amount of 20 pack-years (range 2.5–135 pack-years). Based on the receiver operating characteristic curve analysis, the optimal smoking cut-off value for predicting DFS was defined as 2.5 pack-years, which yielded a distribution of 43 patients (28.9%) in the high smoking group and 106 patients (71.1%) in the low-smoking group (area under the curve: 0.588, sensitivity: 0.325, specificity: 0.846) (Additional file 2: Fig. S2). The ORR was 82.6% and 74 patients (49.7%) achieved a pCR. The therapeutic response was significantly higher among patients with HER2BC than among patients with TNBC (p = 0.023) (Table 3). However, there were no significant differences in the two groups’ pCR rates (p = 0.085), TILs density (p = 0.206), or smoking amount (p = 0.134).

**Table 2:** Clinicopathological features of 149 patients who were treated with preoperative chemotherapy

Parameters (*n *= 149)	Number of patients (%)
Age (years old)	56 (24–75)
Tumour size (mm)	27.6 (10.2–98.0)
Skin infiltration	
Negative/positive	134 (89.9%)/15 (10.1%)
Lymph node metastasis	
N0/N1/N2/N3	51 (34.2%)/54 (36.2%)/29 (19.5%)/15 (10.1%)
HER2	
Negative/positive	87 (58.4%)/62 (41.6%)
Ki67	
Negative/positive	23 (15.4%)/126 (84.6%)
ORR	
Non-responders/responders	11 (7.4%)/138 (92.6%)
pCR	
Negative/positive	75 (50.3%)/74 (49.7%)
Recurrence	
Negative/positive	123 (82.6%)/26 (17.4%)
TILs	
Low/high	58 (38.9%)/91 (61.1%)
Smoker	
No/yes	105 (70.5%)/44 (29.5%)
Pack-years of smokers	20 (2.5–135)
Pack-years	
Low/high	106 (71.1%)/43 (28.9%)

*HER* human epidermal growth factor receptor, *ORR* objective response rate, *pCR* pathological complete response, *TILs* tumour-infiltrating lymphocytes

**Table 3:** Comparison of clinicopathological features by subtype

Parameters	Intrinsic subtype		*p* value
	HER2-enriched breast cancer (*n* = 62)	Triple-negative breast cancer (*n* = 87)	
Age (years old)			
≤ 56	26 (41.9%)	49 (56.3%)	0.085
> 56	36 (58.1%)	38 (43.7%)	
Tumour size (mm)			
≤ 27.6	30 (48.4%)	45 (51.7%)	0.690
> 27.6	32 (51.6%)	42 (48.3%)	
Skin infiltration			
Negative	54 (87.1%)	80 (92.0%)	0.335
Positive	8 (12.9%)	7 (8.0%)	
Lymph node status			
Negative	25 (40.3%)	26 (29.9%)	0.188
Positive	37 (59.7%)	61 (70.1%)	
Ki67			
Negative	14 (22.6%)	9 (10.3%)	0.042
Positive	48 (77.4%)	78 (89.7%)	
ORR			
Non-responders	1 (1.6%)	10 (11.5%)	0.023
Responders	61 (98.4%)	77 (88.5%)	
pCR			
Negative	26 (41.9%)	49 (56.3%)	0.085
Positive	36 (58.1%)	38 (43.7%)	
Recurrence			
Negative	55 (88.7%)	68 (78.2%)	0.096
Positive	7 (11.3%)	19 (21.8%)	
TILs			
Low	20 (32.3%)	38 (43.7%)	0.206
High	42 (67.7%)	49 (56.3%)	
Pack-years			
Low	40 (64.5%)	66 (75.9%)	0.134
High	22 (35.5%)	21 (24.1%)	

*HER* human epidermal growth factor receptor, *ORR* objective response rate, *pCR* pathological complete response, *TILs* tumour-infiltrating lymphocytes

### The associations of smoking with clinicopathological features, DFS, and OS

**Fig. 1:**
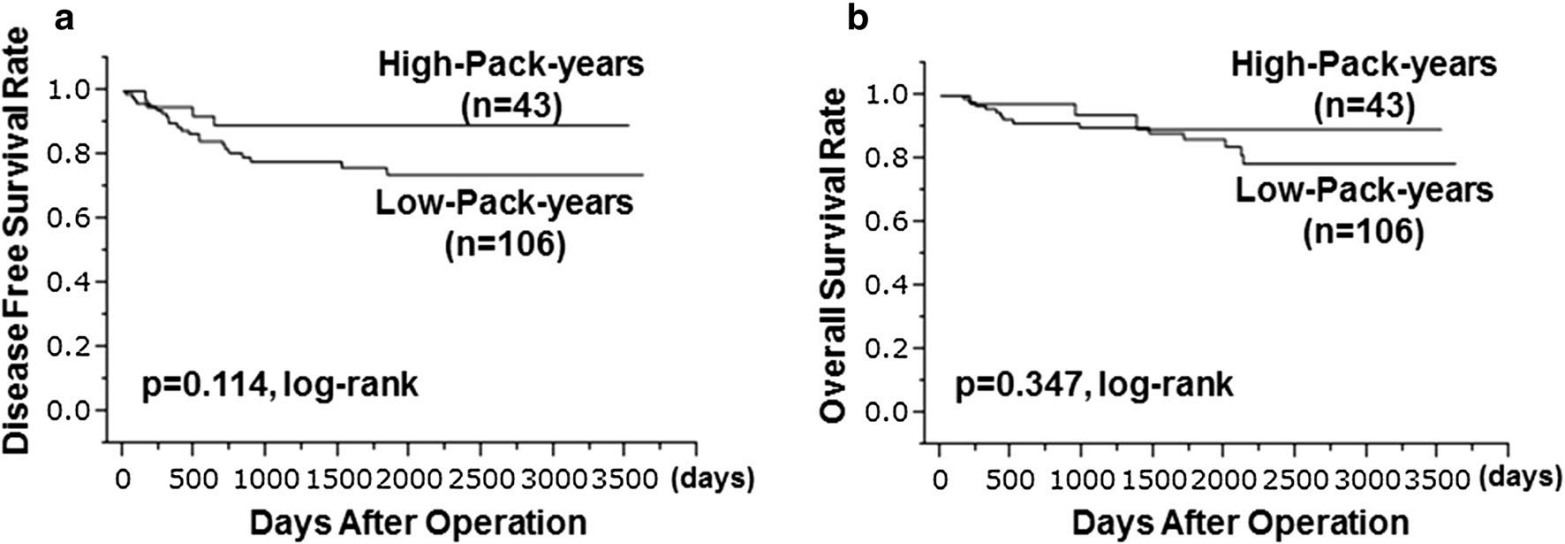
A comparison of disease-free survival (DFS) and overall survival (OS) between the groups with high and low smoking levels failed to detect significant differences in DFS (**a**) or OS (**b**)

Table 4 shows the results of the associations between smoking and the patients’ clinicopathological features. No significant correlation was found between comparing smokers and never smokers. However, when divided into two groups according to smoking amount, correlation with clinicopathological features was recognized. Relative to the low smoking group, the high smoking group had a significant greater TILs density (p = 0.043) and a significantly better pCR rate (p = 0.042). In the univariate analysis, prolonged DFS was significantly associated with pCR (p < 0.001, HR 0.203, 95% CI 0.068–0.499) and a high TILs density (p = 0.001, HR 0.252, 95% CI 0.107–0.553) (Table 5). In addition, prolonged OS was significantly associated with pCR (p = 0.002, HR 0.183, 95% CI 0.042–0.561) and a high TILs density (p = 0.035, HR 0.357, 95% CI 00.129–0.929) (Table 5). However, there was no significant difference according to smoking amount in DFS (p = 0.114) or OS (p = 0.347) (Fig. 1).

**Table 4:** Difference in clinicopathological features due to pack-years

Parameters	Smoker		*p* value	Pack-years		*p* value
	Yes (*n* = 44)	No (*n* = 105)		High (*n* = 43)	Low (*n* = 106)	
Age (years old)						
≤ 56	24 (54.5%)	51 (48.6%)	0.509	24 (55.8%)	51 (48.1%)	0.398
> 56	20 (45.5%)	54 (51.4%)		19 (44.2%)	55 (51.9%)	
Tumour size (mm)						
≤ 27.6	25 (56.8%)	50 (47.6%)	0.309	25 (58.1%)	50 (47.2%)	0.228
> 27.6	19 (43.2%)	55 (52.4%)		18 (41.9%)	56 (52.8%)	
Skin infiltration						
Negative	40 (90.9%)	94 (89.5%)	0.799	40 (93.0%)	94 (88.7%)	0.428
Positive	4 (9.1%)	11 (10.5%)		3 (7.0%)	12 (11.3%)	
Lymph node status						
Negative	18 (40.9%)	33 (31.4%)	0.269	18 (41.9%)	33 (31.1%)	0.214
Positive	26 (59.1%)	72 (68.6%)		25 (58.1%)	73 (68.9%)	
Ki67						
Negative	6 (13.6%)	17 (16.2%)	0.69	6 (14.0%)	17 (16.0%)	0.752
Positive	38 (86.4%)	88 (83.8%)		37 (86.0%)	89 (84.0%)	
Intrinsic subtype						
HER2-enriched	22 (50.0%)	40 (38.1%)	0.181	22 (51.2%)	40 (37.7%)	0.134
Triple-negative	22 (50.0%)	65 (61.9%)		21 (48.8%)	66 (62.3%)	
ORR						
Non-responders	3 (6.8%)	8 (7.6%)	0.866	3 (7.0%)	8 (7.5%)	0.905
Responders	41 (93.2%)	97 (92.4%)		40 (93.0%)	98 (92.5%)	
pCR						
Negative	17 (38.6%)	58 (55.2%)	0.065	16 (37.2%)	59 (55.7%)	0.042
Positive	27 (61.4%)	47 (44.8%)		27 (62.8%)	47 (44.3%)	
Recurrence						
Negative	40 (90.9%)	83 (79.0%)	0.083	39 (90.7%)	84 (79.2%)	0.096
Positive	4 (9.1%)	22 (21.0%)		4 (9.3%)	22 (20.8%)	
TILs						
Low	13 (29.6%)	45 (42.9%)	0.075	12 (27.9%)	46 (43.4%)	0.043
High	31 (70.5%)	60 (57.1%)		31 (72.1%)	60 (56.6%)	

*HER* human epidermal growth factor receptor, *ORR* objective response rate, *pCR* pathological complete response, *TILs* tumour-infiltrating lymphocytes

**Table 5:** Univariate and multivariate analysis with respect to disease-free survival and overall survival

Parameters	Univariate analysis			Multivariate analysis		
	Hazard ratio	95% CI	*p* value	Hazard ratio	95% CI	*p* value
Disease-free survival						
Age at operation (year)						
≤ 56	0.614	0.269–1.335	0.220			
> 56						
Tumour size (mm)						
≤ 27.6	1.137	0.525–2.503	0.744			
> 27.6						
Skin infiltration						
Negative	1.556	0.455–4.067	0.440			
Positive						
Lymph node status						
Negative	2.440	0.933–8.343	0.071	1.677	0.617–5.859	0.331
Positive						
Ki67						
Negative	0.394	0.180–0.926	0.034	0.770	0.321–1.942	0.568
Positive						
Intrinsic subtype						
HER2-enriched	1.884	0.828–4.823	0.135			
Triple-negative						
ORR						
Non-responders	0.083	0.035–0.210	< 0.001	0.154	0.059–0.426	0.001
Responders						
Pathological response						
Non-pCR	0.203	0.068–0.499	< 0.001	0.381	0.118–1.059	0.065
pCR						
TILs						
Low	0.252	0.107–0.553	0.001	0.424	0.167–1.032	0.059
High						
Pack-years						
Low	0.434	0.127–1.134	0.092	0.567	0.160–1.555	0.289
High						
Overall survival						
Age at operation (year)						
≤ 56	0.508	0.175–1.338	0.172			
> 56						
Tumour size (mm)						
≤ 27.6	1.123	0.429–2.993	0.811			
> 27.6						
Skin infiltration						
Negative	1.939	0.446–5.977	0.335			
Positive						
Lymph node status						
Negative	2.778	0.781–17.657	0.125			
Positive						
Ki67						
Negative	0.638	0.234–2.023	0.419			
Positive						
Intrinsic subtype						
HER2-enriched	1.610	0.597–5.060	0.357			
Triple-negative						
ORR						
Non-responders	0.077	0.026–0.238	< 0.001	0.451	0.044–0.520	0.004
Responders						
Pathological response						
Non-pCR	0.183	0.042–0.561	0.002	0.282	0.062–0.953	0.041
pCR						
TILs						
Low	0.357	0.129–0.929	0.035	0.634	0.212–1.861	0.403
High						
Pack-years						
Low	0.554	0.128–1.700	0.325			
High						

*OS* overall survival, *CI* confidence intervals, *HER* human epidermal growth factor receptor, *ORR* objective response rate, *pCR* pathological complete response, *TILs* tumour-infiltrating lymphocytes

## Discussion

Smoking is a risk factor for various carcinomas, including breast cancer [25]. Smoking-related carcinogenesis is linked to various factors, with some of the components in tobacco smoke having estrogenic effects and others having antiestrogenic effects [26, 27]. Moreover, tobacco components can be carried through the blood to the mammary gland tissues where they cause DNA damage [10, 11]. Some researchers have indicated that smoking is associated with the development of ER+ breast cancer, while many others have reported that smoking is associated with ER− breast cancer [26–31]. These differences may be related to race [27], which would be consistent with our findings, as all of our patients were Japanese and had ER− cancers. Furthermore, tissue culture and animal experiments have indicated that tobacco smoke components increase proliferative capacity and cause malignant transformation [32–34], which further highlights the relationship between smoking and the development of TNBC or HER2BC.

The present study indicated that the HER2BC and TNBC subtypes were related to smoking and the cancer’s pre-treatment iTME. Interestingly, previous reports have indicated that a high TILs density was significantly associated with prolonged DFS and OS [4, 5], and the present study indicated that TILs density was associated with the pre-diagnosis smoking amount. These results indicate that local microimmune reactions are activated by chronic inflammation in microvessels, which may be related to the release of antigens as a result of smoking-related DNA damage. Given that a higher smoking amount was associated with a high TILs density, it is possible that smoking was related to the high pCR rate.

Although no previous studies have evaluated the relationship between smoking and the iTME in breast cancer, that relationship has been studied in lung cancer. For example, in non-small cell lung cancer, smoking was not associated with the expression of CD3, CD4, forkhead box protein 3 (FOXP3), and CD20, although smoking was associated with increased CD8 expression [14, 35]. Furthermore, increased numbers of CD8+ T-cells is associated with a good prognosis among patients with non-small cell lung cancer [14, 36]. Moreover, CD8 is a marker for cytotoxic T-cells, which are associated with an improved prognosis among patients with breast cancer [2, 37]. Although the present study did not directly evaluate the correlation between smoking and DFS or OS, the overall exposure to tobacco smoke is known to be associated with the risks of breast cancer recurrence, breast cancer-related death, and overall mortality [38, 39]. In this context, smoking could activate the iTME and affect the short-term therapeutic effect (i.e., pCR rate), although it might not be associated with the long-term therapeutic effect (i.e., DFS or OS) because it is not correlated with low oxygen levels caused by microangiopathy or deterioration of the iTME.

The present study has several limitations. First, the smoking amount was retrospective determined using self-reported data from at the patient’s diagnosis. Second, we did not consider smoking status after diagnosis or second-hand smoke, although passive smoking is an important risk factor for carcinogenesis [25] and lifelong exposure to smoke is more strongly related to the risks of carcinogenesis and recurrence (vs. current smoking status) [38, 39]. It is also reported that special genes, such as MAPKs/TP53, are affecting the iTME [8, 9]. That is, the iTME is also strongly related to genes. Since this result has only been investigated retrospectively, it is necessary to further examine the relationship between smoking and iTME with such as immunohistochemical staining, gene analysis or experiments in vitro. Moreover, it will be important to consider complete smoking-related data to examine the association of smoking with long-term prognosis among patients with breast cancer.

## Conclusions

In conclusion, smoking may influence the iTME, with an activated iTME being associated with pCR rate. Therefore, controlled activation of the microenvironment in this setting may help improve patients’ prognosis.

## Additional files

**Additional file 1: Fig. S1.** Histopathological evaluation of tumor-infiltrating lymphocytes (TILs) density. Specimens were obtained to pathologically diagnose breast cancer using core needle biopsy or vacuum-assisted biopsy, and these specimens were evaluated to calculate the TILs density, which was calculated as the average for five randomly selected stromal regions with lymphoplasmacytic infiltration. (A) > 50%, score 3. (B) 11–50%, score 2. (C) ≤ 10%, score 1. (D) Absent, score 0.

**Additional file 2: Fig. S2.** Receiver operating characteristic curve analysis. The optimal cut-off value for using smoking to predict disease-free survival was identified as 50 pack-years (area under the curve: 0.588, sensitivity = 0.325, specificity = 0.846).

## Abbreviations

iTME: immune tumor microenvironment
TILs: tumor-infiltrating lymphocytes
pCR: pathological complete response
DFS: disease-free survival
OS: overall survival
TNBC: triple-negative breast cancer
HER2BC: human epidermal growth factor receptor 2-enriched breast cancer
POC: preoperative chemotherapy
ER: estrogen receptor
PgR: progesterone receptor
HER2: human epidermal growth factor receptor 2
ORR: objective response rate
pCR: pathological complete response
HR: hazard ratio
CI: confidence interval
REMARK: Reporting Recommendations for Tumor Marker Prognostic Studies
FOXP3: forkhead box protein 3
