# Supplementary figures and images for: Clinical verification of the relationship between smoking and the immune microenvironment of breast cancer
Source: J Transl Med. 2019 Jan 7;17:13. doi: 10.1186/s12967-019-1773-y (PMC6323676; doi:10.1186/s12967-019-1773-y)

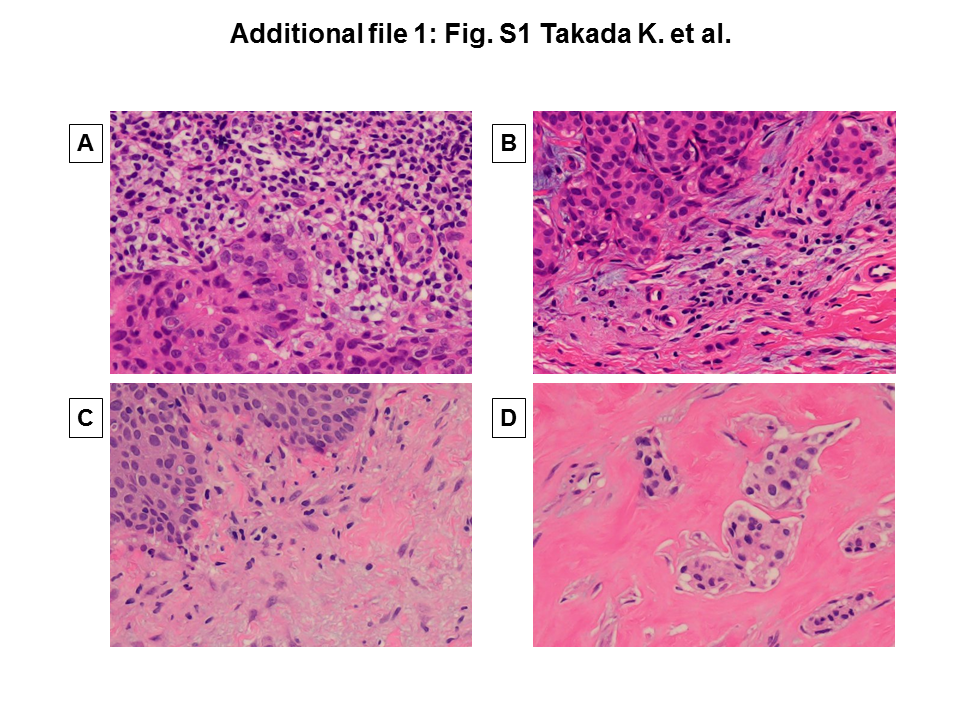

Supplement: Supplementary file 1 — Additional file 1: Fig. S1. Histopathological evaluation of tumor-infiltrating lymphocytes (TILs) density. Specimens were obtained to pathologically diagnose breast cancer using core needle biopsy or vacuum-assisted biopsy, and these specimens were evaluated to calculate the TILs density, which was calculated as the average for five randomly selected stromal regions with lymphoplasmacytic infiltration. (A) > 50%, score 3. (B) 11–50%, score 2. (C) ≤ 10%, score 1. (D) Absent, score 0. [file 12967_2019_1773_MOESM1_ESM.tif]

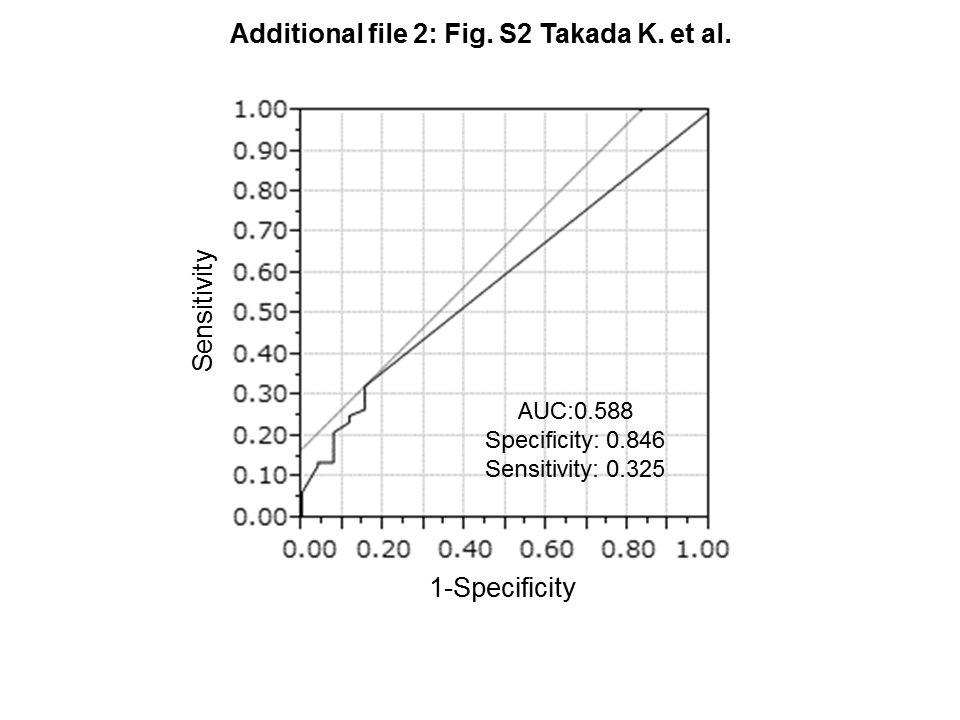

Supplement: Supplementary file 2 — Additional file 2: Fig. S2. Receiver operating characteristic curve analysis. The optimal cut-off value for using smoking to predict disease-free survival was identified as 50 pack-years (area under the curve: 0.588, sensitivity = 0.325, specificity = 0.846). [file 12967_2019_1773_MOESM2_ESM.tif]
